# Therapeutics

**Published:** 1880-04

**Authors:** 


### THERAPEUTICS.

UNALTERABLE COLD-CREAM.—The *Repertoire de Pharmacie* gives the following formula for a cold-cream that possesses the property of not becoming rancid :

| | | | |
|---|---|---|---|
| Quince mucilage | (℥ijss) | 40 | grams. |
| Almond soap | (gr. xv) | 1 | " |
| Stearic acid | (℥ijss) | 10 | " |
| Glycerine | (℥ss) | 2 | " |

EXTERNAL USE OF ATROPINE FOR THE PAIN OF CANCERS.—(*Gaz. Obstet.*, Jan. 20, 1880.)

M. Anger uses, with great success, compresses saturated with a neutral solution of sulphate of atropia, and applied over the seat of pain. The compress is covered with oiled taffeta, or better with sheets of gutta percha, to prevent evaporation, and renewed three or four times a day.

The strength of the solution employed is (grs. xv) 1 gram of the sulphate to (Oij) 1,000 grams of water. He has never seen signs of absorption of the medicine, such as dilatation of the pupil, dryness of the throat, etc. This action is not denied, but it is presumable that the action is local, contraction of the vessels, diminution of the sensibility. The facility of applying it, and the cleanness of this method, give marked advantages over hypodermic injections and ointments. At the same time, the marked relief observed in the terrible pains of cancer seem to recommend its use.

TREATMENT OF SCARLET FEVER.—Archambault. (*Le Praticien*, No. 2, 1880.)

*Hygienic.*—An airy room, no more than the ordinary covering; keep the patient in bed as long as possible, even though the malady seems to have terminated about the seventh or eighth day, when desquamation begins; precautions against taking cold for three or four weeks, for nephritis, which commences from the twelfth to the twenty-third day, rarely comes on after the fourth week. Cover the articulations well, as they are frequently subject to rheumatism or arthritis. The patient should not go out before the sixth week. Baths after the third week. To allay itching, cold-cream and glycerine, or simple dusting with starch powder. Restricted diet at first; more nutritive diet after the fever recedes.

*Medical Treatment.*—If the eruption is long in appearing, borage tea and a potion of (ʒss) 2 grams of acetate of ammonia in (℥iv) 125 grams of mucilaginous mixture. Correct constipation with castor oil or rhubarb. When the eruption is free, give emollient or cooling drinks. If delirium comes on, give bromide of potassium.

Bromide of potassium.........(ʒss–j) 2 to 4 grams.<br>

Syrup of cherry laurel.........(℥ss) 20 grams.<br>

Syrup diacoccymelon.........(ʒijss) 10 "<br>

Hydralate of tilia...............(℥iij) 100 "

of which the child takes a tablespoonful hourly.

*Treatment of the Angina.*—Chlorate of potassium as a gargle, or in younger children in the form of pastilles or mixed with sugar. Caustics are useless. The physician should exercise a rigorous surveillance, even in the simplest cases in appearance, as they may be followed by nephritis, endocarditis, or articular rheumatism.

